# Recent advancement in EUS-guided fine needle sampling

**DOI:** 10.1007/s00535-019-01552-2

**Published:** 2019-02-26

**Authors:** Pujan Kandel, Michael B. Wallace

**Affiliations:** Department of Gastroenterology and Hepatology, Mayo Clinic Florida, 4500 San Pablo Road, Jacksonville, FL 32224 USA

**Keywords:** Endoscopic ultrasound, Fine needle aspiration, Fine needle biopsy

## Abstract

EUS-guided tissue acquisition technique plays an essential role for evaluation of gastrointestinal tumors. Several components affect the yield of EUS-guided tissue acquisition outcomes such as sampling techniques, use of ROSE (rapid onsite evaluation), training and experience, and needle designs. In this review we discuss advancement in EUS-guided fine needle sampling.

## Introduction

Endoscopic ultrasound is one of the important tools for evaluation of gastrointestinal tumors and areas around the gastrointestinal tract. Tissue procurement techniques and tools have evolved significantly over a period of 25 years. Endoscopic ultrasound-guided fine needle aspiration (EUS–FNA) and fine needle biopsy (EUS–FNB) are useful for screening, pathological diagnosis, and staging such as pancreaticobiliary, esophageal, gastric, rectal, and lung diseases [1]. The main end results of EUS-guided fine needle samplings include adverse events, accuracy, histology, and diagnostic yield [2]. EUS-guided tissue sampling is relatively safe and accurate [3]. There are several factors that affect the outcome of this technique such as lesion location and characteristics, experience of endoscopist, EUS fine needle aspiration (FNA) versus fine needle biopsy (FNB), needle gauge, sampling technique, and the presence of onsite cytotechnician [4]. One of the main challenges associated with this technique is low diagnostic yield (false-negative diagnosis) which ranges up to 4%–45% in solid pancreatic mass, 21%–53% in pancreatic cystic neoplasms, and 6%–14% in lymph nodes [5]. The main objective of this review is to focus on the recent advancement in EUS-guided tissue acquisition techniques, needle technologies, and its clinical applications.

## Indication of EUS–FNA

EUS–FNA is useful technique for diagnosis and staging of lesions in and around the proximity of gastrointestinal tract [6–11]. It is superior and effective compared to CT-guided or ultrasound-guided biopsy of lesions [12]. For diagnosis of solid pancreatic lesions, sensitivity and specificity of EUS–FNA is 85%–89% and 96%–99% [13, 14], whereas for pancreatic cystic lesions it is 54% and 93% [15]. The lower sensitivity for diagnosis of cystic lesions is often due to sampling errors, insufficient aspirates, or may be due to different approaches (transgastric or transduodenal) [16, 17]. EUS–FNA is useful technique for pathological diagnosis of abdominal and mediastinal lymph nodes [18]. Tissue procurement from lymph nodes is quite challenging especially in cases of hematologic malignancies such as lymphoproliferative disorders which require adequate tissue to perform immunophenotyping and describe histological architecture. Some studies demonstrated that accuracy of FNA for abnormal lymphocytes is around 70%–90% [19–25]. For mediastinal lymph nodes sensitivity and specificity of EUS–FNA are 88% and 96% vs 84% vs 88% with EUS imaging alone [26]. Thus, EUS–FNA is safer and less invasive compared to mediastinoscopy and is the first choice for small cell cancer staging when combined with bronchoscopy [27, 28]. In addition EUS–FNA is useful for evaluation of sub epithelial lesions, nodal staging of esophageal cancer, liver lesion, and malignant biliary structures [29–31]. Summary of diagnostic values EUS–FNA for different lesions are illustrated below (Table 1). Optimal tissue acquisition from lesions depends on various factors such as needle FNA needle sizes and gauges, fine needle biopsy (FNB) needles, presence of cytotechnologists for rapid onsite evaluation (ROSE), expertise of endoscopist, and tissue handling techniques [32]. American Society for Gastrointestinal Endoscopy (ASGE) guidelines recommend 150 supervised EUS procedures of which 75 should involve pancreaticobiliary system with 50 of them include EUS–FNA [33]. However, recent metaanalysis emphasizes on no clear number of EUS procedures [34] but the success of endoscopist depends on performing EUS at high volume center as procedure is greatly operator dependent [35].

**Table 1:** Diagnostic values for EUS–FNA for different lesions

Lesions	Sensitivity	Specificity	Accuracy
Malignant biliary strictures			
Navaneethan et al. (2015) [117]	66%	88%	–
Sadeghi et al. (2016) [116]	100%	80%	–
Subepithelial lesions (upper and lower GI tract)			
Turhan et al. (2011) [115]	82.9%	73.3%	80%
Larghi et al. (2014) [114]	–	–	93%
Liver lesions			
Tenberge et al. (2002) [31]	–	–	89%
Solid pancreatic lesions			
Hebert-Magee et al. (2013) [113]	88.6%	99.3%	–
Hewitt MJ et al. (2012) [13]	85%	98%	–
Puli SR et al. (2013) [14]	86.8%	95.8%	–
Cystic Pancreatic lesions			
Thronton et al. (2013) [15]	54%	93%	–
Suzuki et al. (2014) [112]	64.8%	90.6%	
Mediastinal lymph nodes			
Puli et al. (2008) [26]	88%	96%	–

## Advancement in EUS-guided FNA and FNB

### Needle gauge and type

For last 2 decades EUS–FNA has been a mainstay for sampling of tissue from pancreas, lymph nodes, liver, and subepithelial lesions. Currently 3 types of EUS-FNA needles are available in market for clinical use: 19G, 22G, and 25G. Several studies have been published comparing 22G vs 25G needles on lymph nodes and solid lesions [36–45]. Most studies showed similar diagnostic yield of malignancy between groups. However, result of the two meta-analyses comparing 25G vs 22G showed that 25G is more sensitive than 22G for diagnosing pancreatic malignancy with overall adequate specimen obtained with 25-gauge needle [46, 47]. Therefore, “*the use of 25G needle is associated with a higher diagnostic yield compared with a 22G needle in patients undergoing EUS*-*FNA of pancreatic masses*” [4]. In addition RCT (randomized controlled trials) have shown no significant difference in diagnostic yield malignancy using 19G needle compared with the 22G/25G needle [48–50]. EUS–FNA technique is relatively safe with few adverse events such as pain, pancreatitis, bleeding, and infections [51] with an morbidity of 0.98%. Pancreatitis rate is about 0.44% and overall mortality is 0.02% [52].

**Fig. 1:**
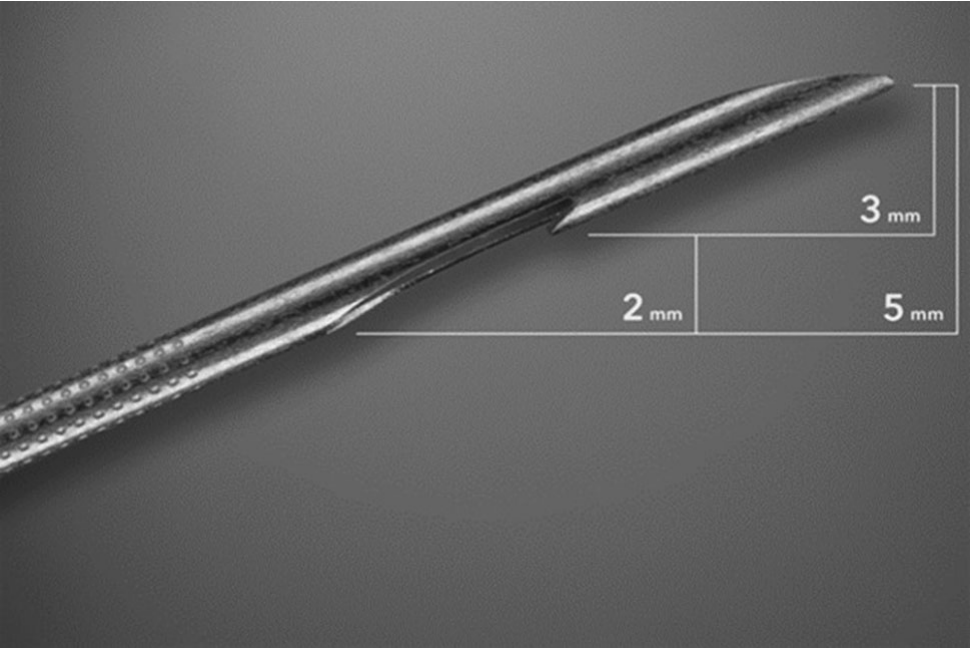
Image of EchoTip ProCore needle tip (22-gauge) showing the reverse bevel that promotes the procuring of core tissue sample from the target lesion (adapted from Dwyer et al. (2016), with permission)

It is often difficult to diagnose with FNA cytology alone in conditions such as autoimmune pancreatitis, lymphoma, and well differentiated adenocarcinoma where histology with preserved tissue architecture is important. Therefore, fine needle biopsy (FNB) is valuable as it provides well-preserved quality tissue samples. Quick-Core (Cook Medical, Limerick, Ireland) was the first FNB needle (also called Tru-Cut core biopsy needle) introduced in market. This 19G needle had an ability to procure large core tissue with a diagnostic yield ranging from 52 to 95% [49, 53–64]. Despite acquisition of large core tissue, it was later removed from marketplace due to lack of its flexibility, availability in only 19-gauge platform, and difficult access through transduodenal route. In addition Quick-Core did not improve diagnostic yield significantly over FNA. This was later replaced by new needle called ProCore (Echo Tip ProCore [ETP], Cook Medical, Limerick, Ireland) with reverse bevel design, Fig. 1. This needle is available in all seizes 19 g, 22 g, and 25 g. The flexibility of this needle allows access through stomach and duodenum without difficulties. Two RCTs have demonstrated similar diagnostic yield of malignancy for FNB and FNA [65, 66]. One recent meta-analysis included nine studies including retrospective, RCTs, and prospective studies [67]. There was no significant difference between ProCore and standard FNA needles in terms of diagnostic adequacy (78% vs 77%), accuracy (86% vs 86%), and core specimen procurement (78% vs 77%) but mean number of passes was significantly lower in ProCore (mean difference 1.2; *p *< 0.01).

**Fig. 2:**
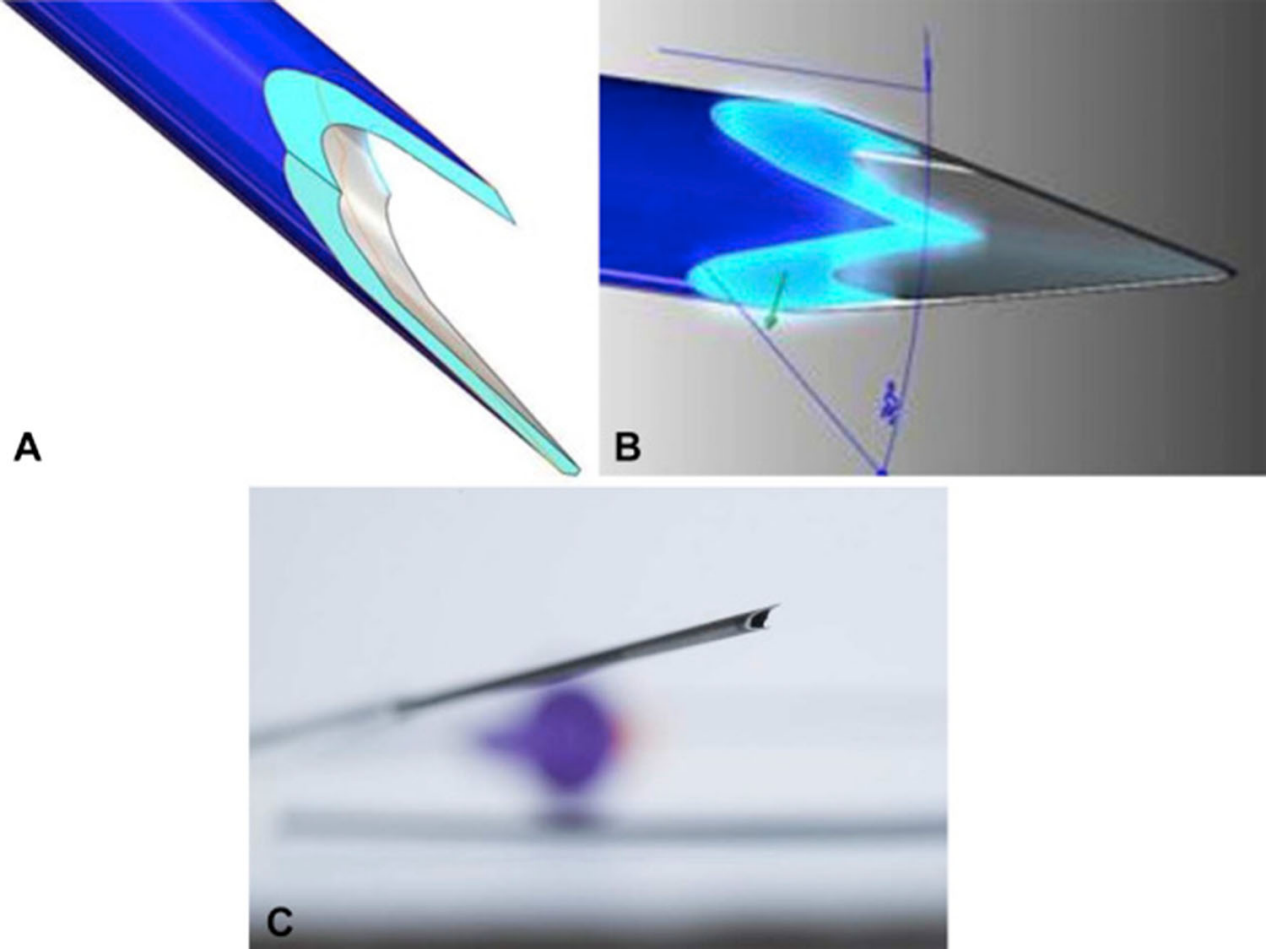
The 22-gauge tip of fork-tip needle with a second tip at opposite side of the lumen (adapted from Kandel et al. (2016), with permission)

**Fig. 3:**
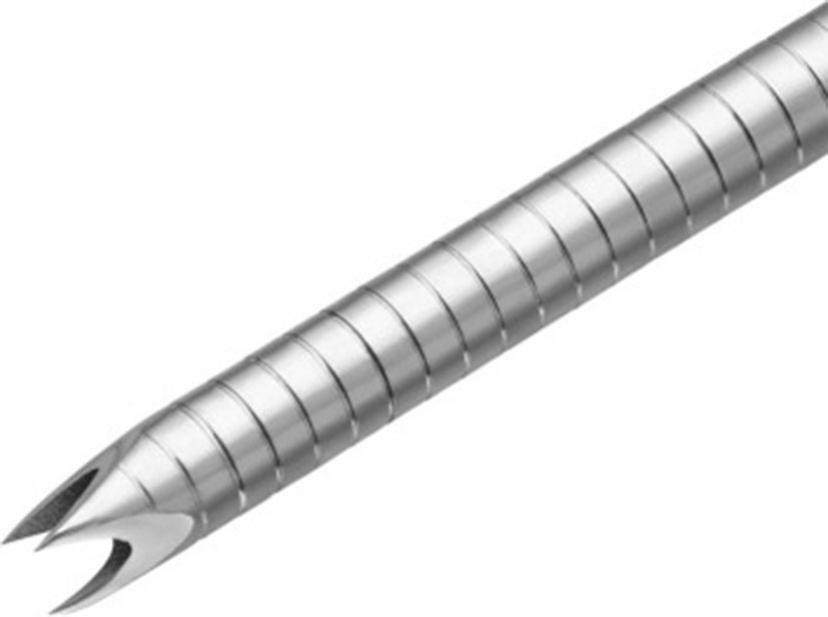
The tip of 22-gauge Franseen needle design with a crown-shaped containing 3 symmetric planes (adapted from Bang et al. (2018), with permission)

In over past 2–3 years, there have been significant paradigm changes in tip of EUS–FNB needle designs. Two FNB needles: one with fork-tip design with two leading sharp tips on the opposite side of the lumen to improve the tissue capture (SharkCore, Medtronic, Minneapolis, Minn.), Fig. 2 and another with three symmetric cutting edges (Acquire, Boston Scientific Corp, Natick, Mass.), Fig. 3 have been introduced in marketplace. Several studies have been published since then and results have demonstrated significant performance in terms of diagnostic yield and histology yield [68–73] for both the needles. In one randomized trial 22-gauge Franseen and fork-tip needles in sampling of solid pancreatic masses were reported. The main outcomes of the study were tumor morphology and histologic adequacy. A total of 50 patients were included in whom sampling was performed using both the needles with the order randomized. Results from this trial showed comparable diagnostic yield (96% vs 92%, *p* = 0.32) and diagnostic adequacy with ROSE (94% vs 96%, *p* = .32) between Franseen and fork-tip needles. In addition there was no statistically significant difference in terms of tissue quality or quantity, total tissue obtained, total tumor tissue, and the desmoplastic fibrosis yield by the two needles [74]. However, in another retrospective study, diagnostic yield was significantly lower in Franseen needle compared to the fork-tip needle (63% vs 77%, *p* = 0.2). In subgroup analysis taking account of only solid pancreatic masses, lower yield was reported with Franseen needle compared to fork-tip needle (64% vs 85%, *p* = 0.01). Therefore, it is more important to have larger multicenter randomized trials to address the fine discrepancies in new FNB needles [75].

Published literatures have demonstrated that FNB is superior to provide adequate histological tissue compared to FNA and may save cost associated with ROSE but the questions still remain: how many dedicated passes are required for different lesions such as gastrointestinal stromal tumor (GIST), solid pancreatic tumor, etc., if to replace ROSE? How safe is FNB? What is the ideal technique? Thus, there is still need of multicenter studies to address these subtle issues.

## EUS-guided tissue acquisition technique

### ROSE (rapid onsite evaluation)

**Fig. 4:**
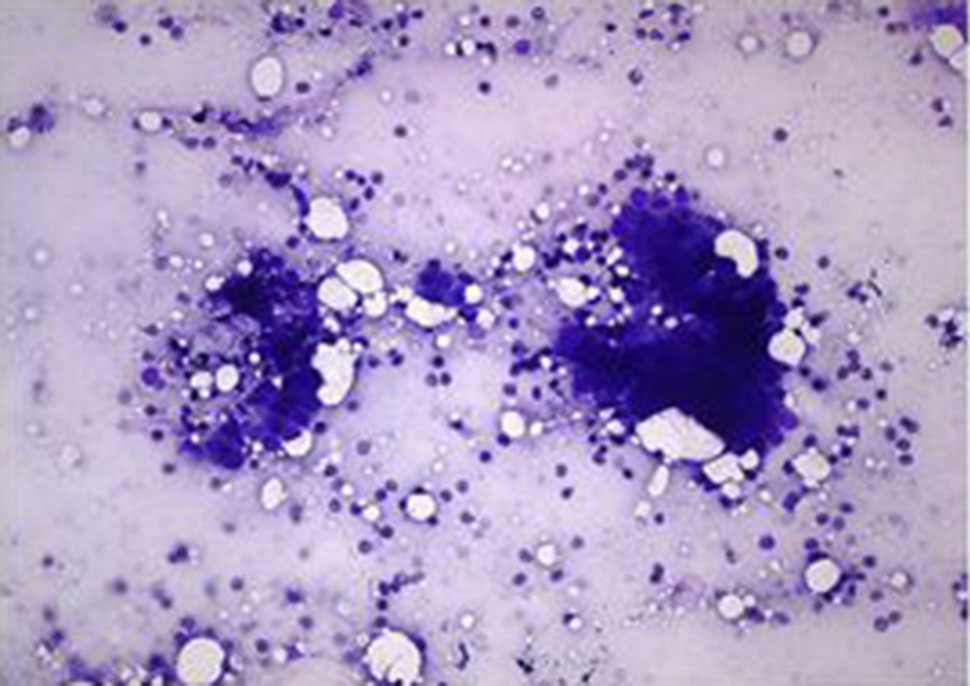
On-site cytological evaluation of pancreatic sample obtained by EUS–FNA supporting the diagnosis of pancreatic adenocarcinoma (adapted from Iglesias-Garcia et al. (2011), with permission)

The main objectives of the ROSE are to provide real time feedback during endoscopy regarding the content and adequacy of specimen, to minimize the number of passes, to decrease inadequate samples, and to increase efficiency of procedure, Fig. 4. In one retrospective study EUS-guided FNA with or without ROSE was compared for sampling of pancreatic mass. Results showed that EUS–FNA with ROSE yielded greater sensitivity (96% vs 78%) and fewer insufficient samples (1% vs 12.6%) with less number of passes [76]. However, results from recent multicenter randomized controlled trial (RCT) showed similar diagnostic yield of malignancy and proportion of inadequate samples when sampling of EUS–FNA of pancreatic masses with and without ROSE. Although the EUS–FNA with ROSE arm required fewer passes, the diagnostic and cytologic yield, adverse events, total procedure time, accuracy, and number of repeat procedures were similar between two groups [77].

With the arrival of new FNB needles, ROSE has less significant impact on diagnostic yield [3, 78–80]. Use of FNB may eliminate ROSE as dedicated core tissue could be obtained with less number of passes. However, in centers with low adequacy rate (< 90%) and less experienced endoscopist, ROSE may have some substantial role [81].

### Stylet and suction use, fanning technique, capillary/slow pull, and wet suction technique

The main aim of using stylet is to prevent blockade lumen of needle as it passes though the gastrointestinal wall. Studies have reported similar diagnostic yield and specimen adequacy with and without stylet [82–84]. In one study flushing of air slowly was superior than insertion of stylet second time to aspirate the samples form solid pancreatic mass [85]. Use of suction yielded higher cellularity, accuracy, and diagnostic yield.

Fanning technique is usually utilized for acquisition of more tissue. One of the studies demonstrated that fanning technique yielded more tissue with fewer number of passes compared to standard technique [86].

Another technique called capillary/slow pull technique is usually utilized to increase aspiration of tissue as it creates negative pressure as stylet is slowly withdrawn during FNA passes. Result from one study demonstrated that adequate and good samples were obtained with capillary technique. However, malignancy yield was similar between suction and capillary technique (90% vs 90%, *p* = 1.00) [86].

**Fig. 5:**
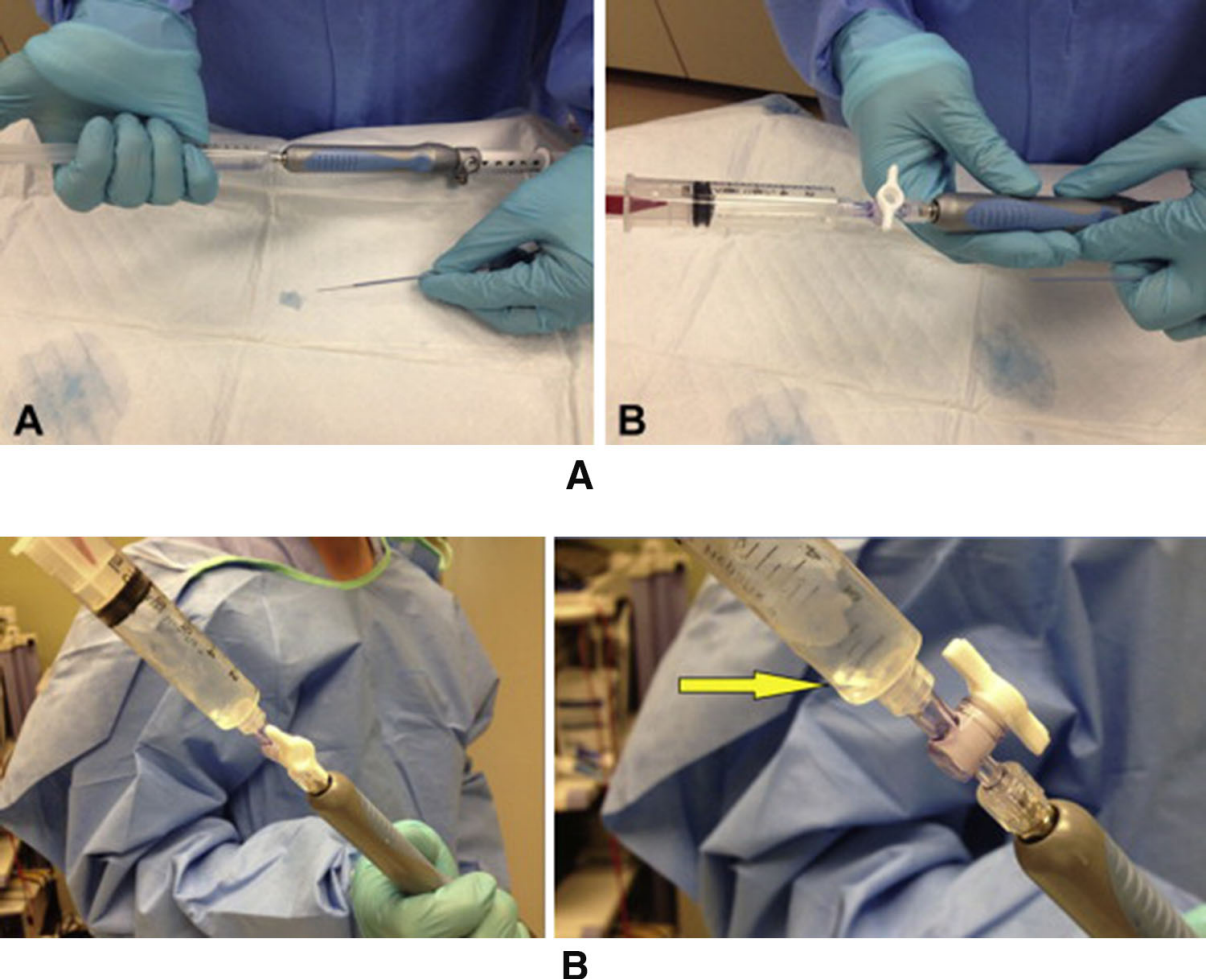
Demonstrating wet suction technique preparation with saline solution (**a**), loading suction syringe in locked position (**b**) and column of saline solution moving into the suction syringe as FNA is performed (adapted from Attam et al. (2015), with permission)

In wet suction technique, air is replaced with saline. Whole idea of this technique to acquire more tissue during EUS–FNA passes. In one RCT wet suction yielded more tissue with higher cellularity compared to standard technique [87], Fig. 5a, b.

### Confocal laser endomicroscopy (CLE) during EUS–FNA

**Fig. 6:**
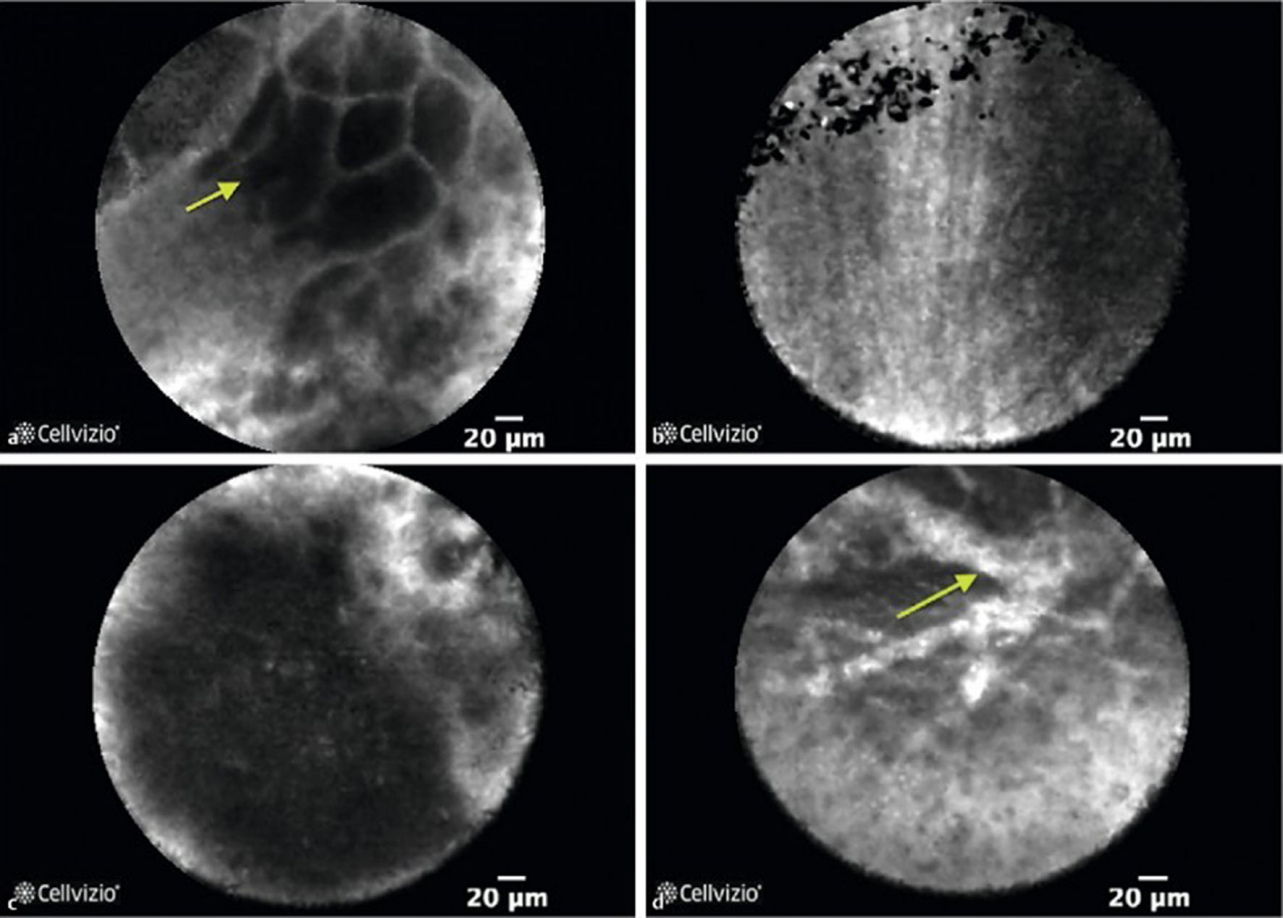
Needle-based confocal laser endomicroscopy (nCLE) images of benign and pancreatic ductal adenocarcinoma lesions. [Benign lesions: **a** showing normal acinar cells, **b**-showing fine white fibrous band representing a fibrotic tissue. Pancreatic ductal adenocarcinoma: **c**- showing dark aggregates > 40 μm, **d**- showing dilated vessels with fluorescein leakage] Adapted from Karstensen et al. (2018), with permission

Confocal laser endomicroscopy is one of the novel imaging technologies that allows microscopic visualization of the mucosal surface epithelium. Optical biopsy at realtime may further improve the diagnostic yield by reducing the sampling error. In addition this may provide the realtime feedback at the time of procedure when onsite cytologist is not available. Needle-based confocal laser endomicroscopy (nCLE) was initially studied in the rat liver and porcine model [88, 89] but the use of this prototype in human solid organs such as pancreas and lymph nodes has been started recently, Fig. 6. This technology involves passage of mini-CLE probe through 19G during EUS–FNA and tissue level can be pictured at realtime. Some studies demonstrated feasibility and safety of nCLE in solid organs [90–92]. In one prospective study, reproducibility and diagnostic value of nCLE for solid lesions were evaluated. Results from this study demonstrated that diagnostic values and interobserver agreement between experts for nCLE parameter were poor [93]. The main limitation of this technology is heterogenicity in histology, interobserver variability, reproducibility, need of pathologists and endoscopists for better interpretation, image quality, and sampling error [94]. Further training and research are needed for applicability in realtime practice.

### EUS-guided needle forceps biopsy

**Fig. 7:**
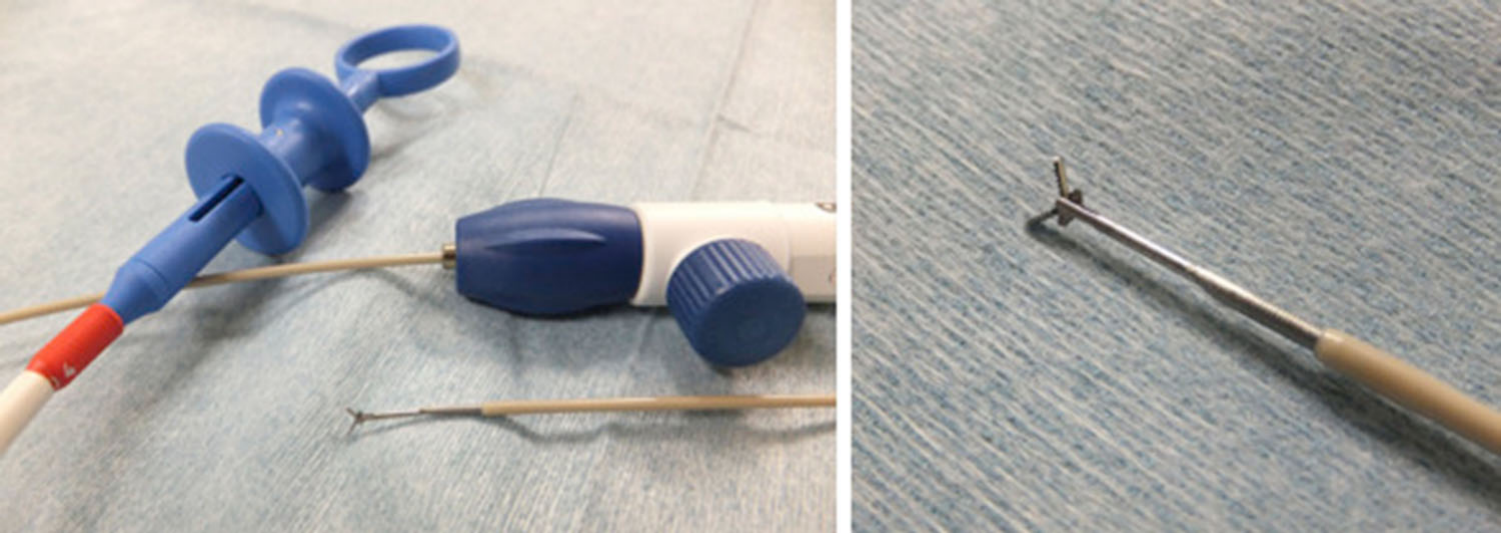
Small biopsy forceps through 19-gauge needle (adapted from Nakai et al. (2016), with permission)

EUS-guided fine needle aspiration is important for cytological diagnosis. Advancement in needle technologies has improved yield of EUS sampling benefiting for both cytological and histological analysis. EUS-guided fine needle forceps biopsy technique was initially started in porcine model [95] which involved the use of small caliber biopsy forceps through 19-gauge FNA needle. In one retrospective study safety and efficiency of EUS-guided through-the-needle forceps biopsy (EUS–TTNFB) were studied. Results from this study demonstrated that EUS–TTNFB was feasible and safe, and provided additional tissue for histological analysis, Fig. 7. No adverse events were reported. Macroscopic core tissue was obtained at a rate of 71% per pass [96].

In addition to solid organs, this novel tissue procurement technique has also been studied in pancreas cystic lesions. Distinguishing the cyst lesions appropriately may help in its management. Cystic lesions that represent diagnostic challenge can be better evaluated histologically with the help of EUS-guided biopsy of the pancreatic cystic walls. Results from one case series demonstrated that needle biopsy forceps was useful in distinguishing the nature of the cyst and stratify their management [97]. Another multicenter retrospective study also evaluated the utility of this technique in evaluation of cystic lesions. The technical and clinical success rate of this technique was found to be 85.7% and 71.4%. Adverse events were observed only in 10.7% of cases [98].

Results from early studies have demonstrated that the use of EUS-guided fine needle biopsy forceps is feasible with reasonable success rates. Multicenter prospective studies are needed to determine its wide spread use in clinical practice compared to other EUS-guided sampling techniques.

### EUS-guided sampling in precision therapy

Precision therapy means providing an individualized management to patient with the use of genomic information [99]. EUS-guided sampling plays a significant role in targeting gastrointestinal cancers in which tissue can be utilized for genomic analysis. This approach increases the therapeutic goal of chemotherapy and reduces side effects associated with chemotherapeutic drug [100]. Studies have reported that cytologic samples obtained by EUS–FNA are an excellent source for genetic analysis [101–105]. However, for years; formalin fixed, paraffin-embedded (FFPE) tissue blocks obtained from surgical samples have been used for genomic analysis.

Recent advancement in needle designs has improved the ability to obtain core biopsies that could be utilized for whole exome sequencing and additional genomic analysis. Development of tumor models such as organoid and xenograft has been possible with core biopsies sample which can be used for in vitro drug testing. Result from one study demonstrated that successful organoid creation was possible in 85% of patients with pancreas cancer [106].

Patient-derived xenografts (PDXs) is created by transmitting the resected tumor tissue from surgery to an immunocompromised host to stimulate the human biology in vivo [100]. This model provides important information on tumor biology that could be utilized for evaluation of different cancers and evaluation of chemotherapeutic drugs [107]. PDXs models can be developed with the use of EUS–FNB technique but delay in engraftment may limit its value in real time management [108].

Newer FNB needle designs have demonstrated adequate core tissue procurement with fewer numbers of passes [109, 110]. More DNA can be extracted from the tumor tissue which could be utilized for full genomic analysis. In one ongoing study by Kandel et al. demonstrated that 74% of EUS–FNB samples of pancreatic cancer were adequate for whole exome sequencing compared to 54% of EUS–FNA samples [111]. Therefore, newer generation needles may be helpful in the era of precision medicine especially in patients with pancreas cancer.

## Conclusion

Advancement in EUS-guided tissue sampling techniques and development of new needle designs that have improved the diagnostic yield of solid lesions. This innovation in EUS has also opened the door for early diagnosis and precision therapy in the management of cancer patients.

## Abbreviation

EUS: Endoscopic ultrasound
FNA: Fine needle aspiration
FNB: Fine needle biopsy
EUS–FNA: Endoscopic ultrasound fine needle aspiration
EUS–FNB: Endoscopic ultrasound fine needle biopsy
RCT: Randomized controlled trial
FFPE: Formalin fixed, paraffin-embedded
GIST: Gastrointestinal Stromal tumor
PDXs: Patient-derived xenografts
EUS–TTNFB: EUS-guided through-the-needle forceps biopsy
ROSE: Rapid onsite evaluation
